# Генная и клеточная терапия функциональных патологий надпочечников: достижения и перспективы

**DOI:** 10.14341/probl12818

**Published:** 2021-12-30

**Authors:** О. В. Глазова, М. В. Воронцова, Л. В. Шевкова, Н. Сакр, Н. А. Онянов, С. А. Казиахмедова, П. Ю. Волчков

**Affiliations:** Национальный медицинский исследовательский центр эндокринологии; Московский физико-технический институт (национальный исследовательский университет); Национальный медицинский исследовательский центр эндокринологии; Московский физико-технический институт (национальный исследовательский университет); Национальный медицинский исследовательский центр эндокринологии; Московский физико-технический институт (национальный исследовательский университет); Московский физико-технический институт (национальный исследовательский университет); Московский физико-технический институт (национальный исследовательский университет), Долгопрудный, Россия; Московский физико-технический институт (национальный исследовательский университет); Национальный медицинский исследовательский центр эндокринологии; Московский физико-технический институт (национальный исследовательский университет)

**Keywords:** надпочечники, стероидогенез, генная терапия, клеточная терапия, органоиды, трансплантация, заболевания надпочечников

## Abstract

Современное понимание организации работы тканей и органов на молекулярном уровне в норме и патологии открывает существенные перспективы для разработки новых, принципиально иных подходов к лечению различных заболеваний. В частности, моногенные наследственные заболевания перестают быть приговором — появившиеся в последнее десятилетие методы геномного редактирования сейчас активно исследуются как инструменты для исправления мутаций в пораженных органах. В свою очередь, появляются новые протоколы получения различных типов клеток и клеточных систем человека и животных, отражающих реальные структуры in vivo. Все эти методы, а также совмещение генной и клеточной терапии активно разрабатываются, и некоторые подходы уже сейчас проходят клинические испытания. Надпочечниковая недостаточность, вызванная разными причинами, также может стать объектом разработки такого рода терапевтических стратегий. Надпочечники представляют собой сложно организованный орган, разные структурные части которого взаимодействуют друг с другом посредством сложной координации эндокринных и паракринных сигналов. В данном обзоре суммированы результаты исследований структурной организации и функционирования надпочечников на молекулярном уровне и современные подходы к лечению патологии надпочечников.

## Abstract (en)

Our current understanding of the molecular and cellular mechanisms in tissues and organs during normal and pathological conditions opens up substantial prospects for the development of novel approaches to treatment of various diseases. For instance, lifelong replacement therapy is no longer mandatory for the management of some monogenic hereditary diseases. Genome editing techniques that have emerged in the last decade are being actively investigated as tools for correcting mutations in affected organs. Furthermore, new protocols for obtaining various types of human and animal cells and cellular systems are evolving, increasingly reflecting the real structures in vivo. These methods, together with the accompanying gene and cell therapy, are being actively developed and several approaches are already undergoing clinical trials. Adrenal insufficiency caused by a variety of factors can potentially be the target of such therapeutic strategies. The adrenal gland is a highly organized organ, with multiple structural components interacting with each other via a complex network of endocrine and paracrine signals. This review summarizes the findings of studies in the field of structural organization and functioning of the adrenal gland at the molecular level, as well as the modern approaches to the treatment of adrenal pathologies.

## ВВЕДЕНИЕ

Кора надпочечников является основным местом синтеза стероидных гормонов, она гистологически и функционально делится на слои, которые окружают центральный мозговой слой.

Прямо под капсулой, которая покрывает всю железу, располагается клубочковая зона (zona glomerulosa, ZG), производящая минералокортикоиды (в основном альдостерон), за ней располагается пучковая зона (zona fasciculata, ZF), синтезирующая глюкокортикоиды (кортизол у человека и кортикостерон у грызунов). У человека также есть сетчатая зона (zona reticularis, ZR), которая синтезирует надпочечниковые андрогены. Производство минералокортикоидов находится под контролем ренин-ангиотензин-альдостероновой системы, в то время как глюкокортикоиды находятся под контролем гипоталамо-гипофизарно-надпочечниковой системы. Глюкокортикоиды регулируют метаболизм глюкозы, воспаление, иммунные реакции, мышечную и скелетную массу, а также познавательные способности, самочувствие и память; в то время как минералокортикоиды контролируют объем внеклеточной жидкости и гомеостаз натрия и, следовательно, имеют важное влияние на кровяное давление [1] (рис. 1).

**Figure:**
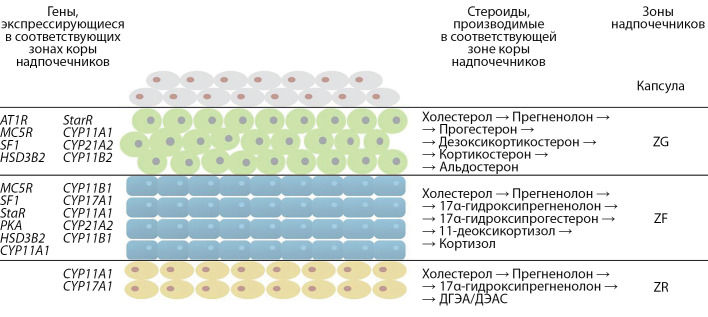
Рисунок 1. Схема строения коры надпочечников человека с указанием зон, а также основных генов и стероидов каждой зоны.

Кора надпочечников — это орган, способный самообновляться на протяжении всей жизни у грызунов (и, предположительно, у людей), заменяя стареющие клетки и поддерживая или расширяя функциональные зоны для удовлетворения физиологической потребности в стероидах или в ответ на внешние фармакологические раздражители [2]. Например, диета с высоким содержанием натрия или лечение ингибиторами ангиотензинпревращающего фермента (АПФ) приводит к уменьшению ZG, и наоборот, диета с низким содержанием натрия приводит к ее увеличению [3]. Лечение синтетическими глюкокортикоидами, такими как дексаметазон, приводит к атрофии ZF из-за снижения уровня адренокортикотропного гормона (АКТГ), вызванного подавлением активности гипоталамо-гипофизарно-надпочечниковой оси (HPA), ZF обычно восстанавливается после прекращения лечения [4].

## ЗАБОЛЕВАНИЯ НАДПОЧЕЧНИКОВ

Надпочечниковая недостаточность проявляется, когда кора не производит адекватные уровни глюкокортикоидов с/без сопутствующего дефицита минералокортикоидов и андрогенов. Болезнь Аддисона (аутоиммунный адреналит) — редкое, но потенциально опасное для жизни эндокринное заболевание, является наиболее частой причиной первичной надпочечниковой недостаточности и возникает в результате двустороннего разрушения коры надпочечников, приводящего к снижению выработки гормонов коры надпочечников, включая кортизол, альдостерон и андрогены [5].

Другой причиной первичной надпочечниковой недостаточности является врожденная гиперплазия коры надпочечников (ВДКН), аутосомно-рецессивное заболевание с общей частотой 1: 9500 новорожденных в РФ [6]. Тяжесть заболевания варьируется от легкой формы, которая может манифестировать во взрослом возрасте, до опасного для жизни состояния при рождении или в раннем младенчестве. Недостаточность возникает в результате мутаций в генах, кодирующих ферменты различных этапов биосинтеза стероидов (CYP11A1, CYP11B1, HSD3B2, CYP17A1). Тем не менее большинство случаев врожденной дисплазии коры надпочечников (ВДКН) (>95%) опосредовано мутациями в гене CYP21A2, который кодирует 21-гидроксилазу (21-ОН) и превращает прогестерон в 11-дезоксикортикостерон и 17-гидроксипрогестерон в 11-дезоксикортизол. При этом выраженность клинического фенотипа коррелирует с типом и расположением молекулярного дефекта. Классическая форма ВДКН характеризуется полной или почти полной потерей активности 21-ОН, что в крайних случаях проявляется жизнеугрожающим сольтеряющим фенотипом. Неклассическая форма ВДКН — менее тяжелый вариант заболевания, проявляющийся в более позднем возрасте андрогенизацией у женщин и бессимптомно у мужчин. На сегодняшний день зарегистрировано более 30 других, в том числе генетических, причин первичной надпочечниковой недостаточности, и механизмы их постепенно выясняются [7].

В настоящее время единственным доступным лечением пациентов с надпочечниковой недостаточностью является заместительная гормональная терапия, и в большинстве случаев она требуется на протяжении всей жизни. Введение экзогенных глюкокортикоидов компенсирует дефицит эндогенного кортизола и действует посредством отрицательной обратной связи с гипоталамусом и гипофизом, подавляя секрецию кортикотропин-рилизинг-гормона и АКТГ. Недостатком традиционной заместительной терапии является то, что подбор корректной дозировки препаратов крайне затруднителен и не воспроизводит физиологический ритм секреции гормонов, а некорректная дозировка при продолжительном лечении может привести к негативным последствиям. К примеру, супрафизиологические дозы экзогенного гормона вызывают ряд побочных эффектов, от легкого подавления гипоталамо-гипофизарной системы до развития сопутствующих инфекционных заболеваний. Длительная терапия низкими и умеренными дозами может вызвать такие проблемы, как ожирение, атрофия кожи, инсулинорезистентность, гипертония и потеря костной массы [8]. Несмотря на то что в последнее время усилия научного и медицинского сообществ были сосредоточены на разработке новых систем доставки глюкокортикоидов, которые могли бы лучше воспроизводить их физиологические уровни, или альтернативных терапевтических подходов, не связанных с глюкокортикоидами [8], коррекция симптомов надпочечниковой недостаточности остается актуальной проблемой.

Принципиально иным подходом к терапии описанных патологий являются генные и клеточные методы лечения. В настоящее время активно разрабатываются и системы доставки инструментов геномного редактирования, и сами подобные инструменты, а также стратегии получения функциональных клеток коры надпочечников in vitro.

## ГЕННАЯ ТЕРАПИЯ

Заболевания, вызванные дефектом одного гена, возможно корректировать с использованием генной терапии. К ним относятся ВДКН, а также более редкий семейный изолированный дефицит глюкокортикоидов (СИДГ), генетическая причина которого выявляется примерно в 67% случаев (мутации в гене рецептора меланокортина типа 2 (MC2R), его белка-партнера, кодируемого геном MRAP1, а также белков, кодируемых генами NNT, StAR и других) [9]. Доступность животных моделей, фенокопирующих клиническую картину ВДКН человека, позволила исследователям протестировать различные варианты доставки генов и зафиксировать восстановление стероидогенеза в ходе экспериментов. В 1988 г. H. Gotoh и соавт. идентифицировали штамм мышей (обозначенный H-2W18) с делецией примерно 80 тыс. пар оснований в области H-2 класса III хромосомы 17, включающей гены компонентов Cyp21a1 и комплемента 4 [10][11]. Мыши, гомозиготные по данной мутации, не обладают активностью 21-ОН и, следовательно, не могут катализировать превращение прогестерона в дезоксикортикостерон, что приводит к их неспособности секретировать кортикостерон (основной биологически активный глюкокортикоид у мышей). Интересно, что гистологический анализ надпочечников у этих мышей выявил нарушение зональности органа: надпочечники имели неправильные очертания, наблюдалась полная дегенерация всех слоев коры, которая сопровождалась хаотичным расположением различных типов клеток, а клетки ZF были увеличены и в размере, и в количестве. Кроме того, у данных мышей гомозиготность напрямую коррелирует с перинатальной летальностью в первые 15 дней жизни [10][12].

Классический путь восстановления дефицита фермента в надпочечниках основывается на использовании вирус-опосредованной доставки генов в дефектную клетку. В 1999 г. T. Tajima и соавт. ввели сконструированный аденовирусный вектор, кодирующий человеческий ген CYP21A2, в надпочечники мышей с дефицитом 21-ОН. Анализ показал наличие мРНК человеческого CYP21A2 в коре и мозговом веществе надпочечников с максимальной экспрессией в первую неделю. Экспрессия гена сохранялась и определялась через 14 дней после введения, хотя была меньшего уровня. Через 2–7 дней после инъекции уровень дезоксикортикостерона и кортикостерона восстанавливался до значений, аналогичных таковым в надпочечниках мышей дикого типа, и сохранялся в течение 40 дней. Кроме того, у этих мышей наблюдали значительное улучшение ультраструктуры клеток надпочечников, о чем свидетельствуют нормальные размеры митохондрий и гистологически нормальные клетки в ZG и ZF [13]. Хотя эффект длился ограниченное количество дней, данное исследование показало, что рекомбинантные аденовирусы можно использовать в качестве векторов для доставки трансгенов путем прямого введения в надпочечники.

Первичные фибробласты, трансдуцированные геном Cyp21a1 мыши с использованием ретровирусного вектора, были трансплантированы в подкожные ткани 21-ОН-дефицитных мышей: эта процедура привела к снижению соотношения прогестерон/дезоксикортикостерон в сыворотке у 4 из 6 животных через 4 нед после аутотрансплантации. Авторы признали потенциал такого метода лечения, но вместе с тем объясняют не полностью удовлетворительный результат либо недостаточным количеством инфицированных фибробластов, либо низким титром вирусного вектора [14].

Параллельно другой группой ученых был проведен эксперимент с инъекцией в мышцы бедра аденоассоциированного вируса серотипа 2 (AAV2), содержащего ген Cyp21a1, что также привело к значительному снижению соотношения прогестерон/дезоксикортикостерон в сыворотке крови, причем на этот раз у 100% обработанных мышей. Показатель соотношения оставался относительно низким в течение 7 мес после инъекции. Несмотря на небольшой размер выборки, эта работа впервые показала, что внеадреналовая индукция активности 21-ОН улучшает системный метаболизм стероидов у мышей с ВДКН и что менее инвазивные методы индукции генов также возможны [14].

Другой многообещающий результат был получен M. Perdomini и соавт. с использованием AAV серотипа rh10 (AAVrh10), уже применяемого в исследованиях генной терапии на человеке. Внутривенная инъекция AAVrh10, несущего человеческий ген CYP21A2, взрослым 21-ОН-дефицитным мышам приводила к экспрессии 21-ОН и почти нормальной экспрессии ключевых генов (Mc2r, Prkar2a, Sf-1, Star, Cyp17a1 и Cyp11b2) в коре надпочечников. Важно отметить нормализацию ранее повышенного уровня прогестерона, что указывает на значительное и устойчивое восстановление активности 21-ОН, сохраняющейся в течение 15 нед [15].

В относительно недавнем исследовании был проведен более подробный анализ наблюдаемой кратковременной эффективности доставки CYP21A2 в клетки надпочечников. Однократное внутривенное введение AAVrh10, несущего человеческий ген CYP21A2, мышам с дефицитом 21-ОН позволило нормализовать уровни прогестерона и АКТГ между 2-й и 8-й неделями после инъекции, но показатели обоих гормонов снова повысились, достигнув исходных уровней через 32 нед. Интересно, что иммуногистохимический анализ гемагглютининовой метки, связанной с С-концом 21-ОН, показал экспрессию высокой интенсивности трансгена в ZF- и в X-зоне, но не в ZG-зоне и не в клетках капсулы. Более того, анализ трансдуцированных клеток с течением времени показал прогрессивное снижение экспрессии трансгена через 6 и 16 нед после введения вплоть до почти полного отсутствия трансдуцированных клеток через 32 нед [16]. Как заключают авторы исследования, весьма вероятно, что причина временной эффективности такого подхода кроется в биологии коры надпочечников и ее непрерывном самообновлении. Капсула и ZG содержат по крайней мере две популяции адренокортикальных стволовых клеток/клеток-предшественников. Если эти клетки не будут эффективно трансдуцированы (то есть в их геном не будет введен необходимый ген), то со временем стероидогенные потомки нетрансдуцированных клеток будут заменять более старые функциональные (трансдуцированные) клетки ZF, что в итоге приведет к восстановлению фенотипа ВДКН и нивелированию результатов лечения. Авторы пришли к выводу, что, хотя специфические векторы типа AAV, использованные в эксперименте, а также классическая генная терапия эффективны в краткосрочной перспективе, данный подход не позволяет добиться долгосрочных результатов. Следовательно, для достижения пожизненного терапевтического эффекта важно наличие таких серотипов AAV, которые способны трансдуцировать адренокортикальные стволовые и прогениторные клетки. Вместе с тем существование AAV, наделенных таким тропизмом, еще предстоит определить экспериментально, а также могут потребоваться другие методы доставки [17]. Кроме того, возможно, понадобятся доставка не только здоровой копии самого гена, но и инструменты для его встраивания в геном клетки.

Альтернативной стратегией доставки полноразмерных открытых рамок считывания могло бы быть применение инструментов редактирования генома в адренокортикальных стволовых клетках, что позволило бы целенаправленно корректировать дефекты генов как в стволовых клетках, так и в стероидогенных дифференцированных их потомках. Подход, основанный на редактировании генов, был успешно применен в мышиных моделях мышечной дистрофии [18–20]. Кроме того, используя двойную систему, где один AAV8 вектор экспрессировал нуклеазу Cas9, а второй вектор того же серотипа экспрессировал направляющую РНК и матрицу ДНК донора для репарации, Y. Yang и соавт. смогли исправить точечную мутацию в гене орнитин-транскарбамилазы в печени на модели метаболического заболевания печени у новорожденных мышей. Важно отметить, что эта работа показала возможность генной коррекции в регенерирующих тканях [21].

Ввиду сложной организации геномного локуса, в котором расположен ген CYP21A2, наиболее эффективной стратегией предположительно является доставка и интеграция в геном целой кодирующей последовательности CYP21A2 дикого типа. Другие препятствия, которые необходимо учитывать при разработке генотерапевтических подходов — это потенциальная онкогенность вирусных векторов, небольшая упаковочная способность и иммунный ответ хозяина на вирусный вектор, короткая продолжительность экспрессии доставленной здоровой копии гена и многие другие [22]. Наконец, возможной альтернативой вирусным векторам могут быть системы доставки генов на основе невирусных плазмид: их можно вводить повторно, они обладают низкой токсичностью и иммуногенностью. Кроме того, постоянно разрабатываются новые способы улучшения переноса плазмидной ДНК в ядра клеток [17].

## КЛЕТОЧНАЯ ТЕРАПИЯ

В последние годы методы лечения на основе клеток продемонстрировали большой терапевтический потенциал в рамках доклинических исследований на моделях животных различных патологических состояний, показав себя как уникальный инструмент для восстановления или замены поврежденных тканей. Конечной целью данных испытаний являлись регенерация и восстановление нормальной функции органов и тканей. В эндокринологии усилия научного сообщества привели к первым клиническим испытаниям с использованием поджелудочной железы, полученной путем дифференцировки эмбриональных стволовых клеток (ЭСК) и индуцированных плюрипотентных стволовых клеток (ИПСК), для лечения диабета 1-го типа [23]. Использование стратегий клеточной терапии для создания функциональной ткани коры надпочечника является многообещающей альтернативой существующим методам лечения надпочечниковой недостаточности вне зависимости от этиологии заболевания, поскольку такой подход позволяет сформировать более физиологичное функционирование органа.

Хотя разработка стратегий клеточной терапии в области надпочечников находится в начальном своем пути, важно признать, что на сегодняшний день было получено несколько значимых результатов. В следующих разделах будут обсуждаться современные подходы и достижения, включая применение клеток надпочечников для трансплантации, использование функциональных стероидогенных клеток, полученных из стволовых клеток или репрограммированных соматических клеток, а также создание объемных моделей адренальных органоидов.

## ТРАНСПЛАНТАЦИЯ КЛЕТОК НАДПОЧЕЧНИКОВ

В предшествующие годы были разработаны различные протоколы выделения и ограниченной пролиферации клеток надпочечников для алло- или ксенотрансплантации. Были изучены различные стратегии, типы клеток и животные модели с акцентом как на эффективную компенсацию трансплантированными клетками необходимого функционала, так и на долгосрочную безопасность такой процедуры. M. Thomas и соавт. изолировали клетки надпочечников крупного рогатого скота путем ферментативного и механического диспергирования коры надпочечников. Затем под капсулу почки мышей с тяжелым комбинированным иммунодефицитом (severe combined immunodeficient mice, SCID) инъецировали цилиндр из поликарбоната, который был заполнен адренокортикальными клетками крупного рогатого скота. Кроме адренокортикальных, присутствовали также непролиферирующие клетки 3T3, которые секретировали факторы роста фибробластов (fibroblast growth factor, FGF) с целью усиления васкуляризации. Пересаженные бычьи клетки формировали надпочечниковую ткань, способную выполнять основные функции собственных мышиных надпочечников, о чем свидетельствует выработка стабильных уровней кортизола [24][25].

Затем та же группа ученых адаптировала свой протокол к клеткам коры надпочечников человека, выделенных из ZF. Клетки культивировали в течение 5–7 дней перед трансплантацией SCID-мышам, как было описано ранее. Через 50 дней после трансплантации гистологический и иммуногистохимический анализы показали, что полученная ткань имела характеристики нормальной коры надпочечников, а кортизол присутствовал в плазме животных в ответ на стимуляцию АКТГ. Таким образом, было продемонстрировано, что адренокортикальные клетки человека могут быть использованы для замены функции надпочечников у животного-реципиента [26].

Вышеупомянутые исследования побудили к дальнейшей работе, направленной на оптимизацию процедур трансплантации надпочечников. Например, различными группами исследователей было протестировано использование вспомогательного материала, имитирующего внеклеточный матрикс коры надпочечников для облегчения трансплантации и приживания клеток путем обеспечения для них необходимого окружения. N. Popnikolov и P. Hornsby встроили нормальные клетки коры надпочечников человека/крупного рогатого скота в матрицу из коллагена I типа перед подкожной трансплантацией этих клеток SCID-мышам. Было показано формирование узелков с инвазией новообразованной сосудистой сети, а также областей с характерной для ZF гистологической структурой. Кроме того, в плазме мышей-реципиентов был обнаружен кортизол [27]. J. Dunn и соавт. культивировали диссоциированные клетки надпочечников новорожденных мышей на губке из бычьего коллагена перед трансплантацией в почечную капсулу взрослых мышей. Анализ мРНК имплантата показал экспрессию специфичных для надпочечников маркеров (Sf-1, Dax1, Star, Cyp11a, Cyp11b1 и Cyp21a2) в течение периода до 8 нед после трансплантации [28]. Впоследствии, используя эту технику, группа ученых смогла достичь 100% выживаемости мышей, перенесших двустороннюю адреналэктомию. Более того, уровень кортикостерона в плазме животных был сравним с таковым у контрольных образцов, а анализ извлеченных имплантатов показал наличие жизнеспособных клеток и экспрессию адренокортикальных генов, хотя полноценно сформированных желез не наблюдалось [29].

В другой работе в качестве каркаса для эмбриональных клеток надпочечников человека использовались децеллюляризованные надпочечники свиней: анализ поведения клеток in vitro показал, что они способны прикрепляться к такому каркасу, пролиферировать и продуцировать кортизол до уровня, сопоставимого с литературными значениями для нативных тканей [30]. В еще одной работе M. Balyura и соавт. разработали протокол создания искусственных надпочечников из первичной культуры бычьих адренокортикальных клеток, инкапсулированных в альгинатный матрикс. Преимущества использования альгинатов заключаются в том, что они снижают риск иммунного ответа хозяина, таким образом обеспечивая трехмерную структуру, которая облегчает межклеточное взаимодействие. Пересадка инкапсулированных клеток способствовала активной васкуляризации трансплантата, предотвращала смертность, вероятность которой высока при двусторонней адреналэктомии, а также более эффективно восстанавливала уровень глюкокортикоидов в сравнении с процедурой трансплантации свободных клеток [31].

Очищенные клетки ZG и ZF, полученные из надпочечников крыс путем разделения в градиенте плотности, также были использованы в качестве исходного материала для трансплантации под капсулу почки адреналэктомированных крыс. Часть клеток была пересажена непосредственно после подготовки, часть после прохождения процесса культивирования. Было замечено, что через 30 дней внедренные под капсулу почки клетки ZG, в отличие от клеток ZF, были способны образовывать тканевую структуру, гистологически напоминающую ZG. Кроме того, они продуцировали альдостерон и кортикостерон, что делает их более подходящими для трансплантации, чем клетки ZF [32]. Принимая во внимание наличие пула клеток-предшественников надпочечников именно в ZG крысы [33] и их способность трансдифференцироваться в клетки ZF [34], можно предположить, что именно эти субпопуляции были ответственны за высокий потенциал дифференцировки трансплантированных клеток, обеспечив успешное формирование ткани у животного-реципиента.

В другом исследовании была предпринята попытка выделить мышиные предшественники коры надпочечников из дифференцированных клеток на основе неодинакового сродства к флуоресцентному красителю Nile red, который взаимодействует с холестерином, локализованным в цитоплазме (высокое содержание в дифференцированных клетках и низкое/нулевое в недифференцированных клетках). Были идентифицированы две группы клеток: более светлые окрашенные клетки (с низким сродством к Nile red), которые экспрессировали мРНК Sf-1 на уровнях, сопоставимых с более ярко окрашенными клетками (с сильным сродством к Nile red), но значительно более низкими уровнями Cyp11b1, Cyp11b2 и мРНК Cyp11a1. При имплантации мышам менее окрашенные клетки смогли дать начало клеткам, экспрессирующим зонально-специфические гены коры надпочечников, в отличие от более ярко окрашенных клеток, которые утратили экспрессию стероидогенных ферментов и свой потенциал к пролиферации. Авторы предположили, что данный метод окрашивания помог выявить ту популяцию клеток (светлоокрашенные), что была обогащена клетками-предшественниками надпочечников, которые, в свою очередь, дали начало популяции стероидогенных клеток [35]. Данное предположение встраивается в актуальное понимание о самообновлении надпочечников [33].

Дальнейшие эксперименты в этой области направлены на повышение эффективности трансплантации клеток. Было показано, что экспансия клонов клеток in vitro, иммортализованных с использованием обратной транскриптазы теломеразы человека (hTERT), формирует васкуляризированные тканевые структуры при трансплантации под капсулу почки, которые способны восстановить функцию надпочечников животных. Важно отметить, что модифицированные hTERT клетки не демонстрируют тенденции к неопластическим изменениям [36].

Плодотворное сотрудничество между специалистами разных областей науки и медицины оказалось абсолютно необходимым для ускорения разработок в этих областях, примером чего является получение новых герметизирующих устройств, изготовленных из различных материалов, которые предлагают хорошую альтернативу рассмотренным выше матриксам для трансплантированных клеток надпочечников. Некоторые из этих полупроницаемых и иммуноизолирующих устройств, загруженных панкреатическими клетками или β-клетками, в настоящее время проходят испытания на пациентах с сахарным диабетом 1-го типа. Тем не менее эти исследования еще не дали полностью удовлетворительных результатов. Например, состояние нормогликемии было достигнуто лишь в нескольких случаях, секреторная способность трансплантированных клеток ограничена, а безопасность и переносимость этих устройств находятся на стадии оживленных дискуссий [37].

## ДИФФЕРЕНЦИРОВКА СТВОЛОВЫХ КЛЕТОК IN VITRO

Стволовые клетки обладают способностью к самообновлению и могут дифференцироваться в определенные типы клеток, что делает их идеальным кандидатом для исследований в регенеративной медицине. Вместе с тем открытие, что зрелые полностью дифференцированные клетки можно репрограммировать и трансформировать в клетки другого типа, стало значительным прорывом и дало уникальный источник клеток для аутологичной клеточной заместительной терапии. В 2006 г. ученый C. Яманака продемонстрировал, что дифференцированные мышиные клетки могут быть возвращены в плюрипотентное состояние, схожее с эмбриональными стволовыми клетками, путем активации экспрессии генов, которые кодируют четыре фактора транскрипции [38]. В правильных условиях ИПСК могут дифференцироваться в разные специализированные типы клеток.

Наиболее часто используемым ресурсом для получения стероидогенных клеток на сегодняшний день являются мезенхимальные стромальные клетки (МСК). В ранних экспериментах было показано, что после индукции циклическим аденозинмонофосфатом (цАМФ) и третиноином клетки, стабильно экспрессирующие белок SF-1, но не нативные мышиные эмбриональные стволовые клетки (мЭСК), экспрессировали мРНК P450Scc и продуцировали прогестерон [39]. Эта экспериментальная стратегия была улучшена спустя годы учеными Yazawa и соавт., которые смогли дифференцировать мЭСК в мезенхимальные клетки путем культивирования клеток на чашках, покрытых коллагеном IV, и импульсного воздействия ретиноевой кислоты. Впоследствии форсированная экспрессия SF-1 привела к экспрессии различных генов, связанных со стероидогенезом, и к секреции кортикостерона, подобно тому, как это происходит в адренокортикальных клетках [40].

Схожий подход был успешно применен для дифференцировки человеческих ЭСК в мезенхимные клетки (на этот раз с использованием ингибитора киназы-3β гликогенсинтазы). Далее, при индукции экспрессии SF-1 и цАМФ были получены гормон-продуцирующие клетки [41]. S. Gondo и соавт. продемонстрировали, что первичные длительно культивируемые стволовые клетки костного мозга мыши (bone marrow cells, BMC), инфицированные аденовирусной конструкцией, которая содержит бычий SF-1, экспрессируют стероидогенные гены и продуцируют значительное количество стероидов (хотя экспрессия Cyp11b2 и альдостерон не были обнаружены). Тем не менее стероидный профиль показал смешанную картину стероидогенеза надпочечников и гонад. Интересно, что даже в условиях отсутствия специальной индукции экспрессии рецепторов к АКТГ ответ клеток в виде активации стероидогенных генов и гормонов все же усиливается в ответ на введение тропного гормона [42]. Совсем недавно было показано, что индуцированные SF-1 стероидогенные клетки, полученные из МСК жировой ткани мышей, повышают базальный уровень кортикостерона в плазме и продлевают выживаемость мышей, подвергнутых двусторонней адреналэктомии [43].

В рамках аналогичного протокола авторы расширили свою работу и использовали мезенхимальные клетки, выделенные из жировой ткани (adipose mesenchymal cells, AMC). Форсированная экспрессия SF-1 трансформировала АМС в АКТГ-чувствительные стероидогенные клетки, способные производить кортикостерон надпочечников, а не тестостерон, в отличие от BMC. Важно отметить, что добавление третиноина усилило сдвиг стероидного профиля в сторону надпочечникового типа в AMC по сравнению со стероидным профилем гонадного типа в BMC. Это наблюдение указывает на то, что источник клеток и условия культивирования являются важными факторами в определении пути дифференцировки [44].

В то же время T. Yazawa и соавт. добились положительных результатов с использованием МСК человеческого происхождения. В их исследовании стабильная экспрессия SF-1 привела к увеличению производства клетками большинства стероидогенных ферментов и выработке стероидов надпочечников и гонад в присутствии цАМФ [45]. Позже было показано, что ретровирус-опосредованная экспрессия гомолога-1 рецептора печени (LRH-1), другого члена семейства ядерных рецепторов NR5A и высоко экспрессируемого в гонадах человека, индуцирует дифференцировку МСК человека в стероидогенные клетки, что представляет собой возможную альтернативу для достижения стероидогенного фенотипа [46].

Дифференцировка МСК пуповинной крови (UCB-MSC) или МСК из Вартонова студня (UC-MSC) также оказалась успешной. В 2010 г. T. Yazawa и соавт. показали, что UCB-MSC могут быть использованы для получения стероидогенных клеток посредством ретровирусной трансдукции геном SF-1, но демонстрируют заметно отличающиеся стероидогенные профили, скорее гонадного типа, в сравнении с клетками, полученными из BM-MSC [47]. С другой стороны, X. Wei и соавт. сочли UC-MSC более подходящим клеточным субстратом, поскольку после дифференцировки они показали более высокий пролиферативный потенциал и значительно более высокую экспрессию всех протестированных стероидогенных мРНК [48].

Использование ИПСК для генерации стероидогенных клеток на сегодняшний день находится в менее разработанной стадии в сравнении с другими источниками стволовых клеток, однако существующие исследования демонстрируют перспективность этого направления. В 2012 г., следуя многоступенчатому протоколу, T. Sonoyama и соавт. получили стероид-продуцирующие клетки из фибробластов кожи человека, репрограммированных в ИПСК. Далее полученные ИПСК дифференцировали в клетки мезодермального пути в присутствии 6-броминдирубин-3’-оксима, ингибитора гликогенсинтазы киназы-3-β, что приводило к активации пути передачи сигнала Wnt. В конечном итоге принудительная экспрессия SF-1 в этих мезодермальных клетках и добавление 8-Br цАМФ привели к образованию клеток, которые экспрессировали мРНК CYP21A1, CYP11B1, StAR, CYP11A1, HSD3B и CYP17A1 и секретировали кортизол [41].

Недавно также было продемонстрировано, что клетки крови, кожи и мочи можно дифференцировать с получением индуцированных стероидогенных клеток (hiSCs). Это было достигнуто за счет лентивирус-опосредованной экспрессии SF-1 и активации пути протеинкиназы A в присутствии гонадотропин-рилизинг-гормона [49]. Полученные клетки экспрессировали стероидогенные ферменты и секретировали кортизол при наличии стимула. Кроме того, эксперименты in vivo продемонстрировали жизнеспособность этих клеток после трансплантации в надпочечники и под капсулу почек мышей. Более того, стероидный профиль hiSCs, полученных из клеток мочи пациентов с мутациями CYP21A2, показал накопление субстратов 21-ОН и снижение продуктов гидроксилирования по сравнению с клетками здоровых доноров, таким образом демонстрируя патологический фенотип ВДКН. Критически важно, что восстановление нормального стероидогенеза этих клеток может быть достигнуто путем доставки и экспрессии в них здоровой копии гена [49]. Этот результат особенно важен, поскольку он свидетельствует о том, что hiSCs могут представлять собой отправную точку для создания новых экспериментальных моделей ВДКН и разработки новых пациент-специфичных методов лечения.

Необходимо учитывать, что для развития терапевтических подходов необходимо создание надежных протоколов для получения адренокортикальных клеток, способных к самообновлению, где должен быть учтен ряд условий, а именно: тип и источник клеточного субстрата, компоненты среды, молекулярные индукторы, а также возможности последующего контроля качества результирующей клеточной культуры.

Нужно учитывать, что экзогенно привнесенная принудительная экспрессия факторов дифференцировки, которая не подвержена естественной регуляции со стороны клетки, приводит к формированию неспецифического клеточного фенотипа, часто смешанному гонадо-адреналовому. В это же время работа ряда других естественных регуляторных путей в трансфицированных клетках не в полной мере соответствует нормальным клеточным типам органа. В настоящее время наиболее корректными считаются протоколы, основанные на использовании малых молекул (BMP, FGF, Wt и др.), имитирующих эмбриональное развитие клеточного типа и запускающих необходимые пути для корректной дифференцировки в нужный клеточный тип. Ввиду вышесказанного, а также учитывая ключевую роль SF-1 в виде эндогенного фактора транскрипции, участвующего в развитии и надпочечников, и гонад, создание поэтапного протокола получения клеток коры надпочечников без принудительной экспрессии какого-либо фактора транскрипции извне было бы ценным научным прорывом в области дифференцировки клеток надпочечников.

## ТРЕХМЕРНЫЕ МОДЕЛИ И ОРГАНОИДЫ НАДПОЧЕЧНИКОВ

В последние годы плюрипотентные стволовые клетки, взрослые стволовые клетки/клетки-предшественники или эмбриональные клетки-предшественники были использованы для разработки моделей органов человека in vitro, известных как органоиды, которые более точно воспроизводят клеточную структуру и взаимодействие в различных тканях органов. Органоиды — это трехмерная многоклеточная структура, полученная из резидентных стволовых клеток целевой ткани, которые дифференцируются и самоорганизуются, а также способны воспроизводить многие ключевые структурные и функциональные особенности органа [50]. Разработка протоколов создания органоидов станет значимым дополнением к существующим методам исследований функциональных нарушений надпочечников, знания о которых в основном были получены путем изучения мышиных моделей. Присутствие стволовых клеток/клеток-предшественников в капсуле/субкапсулярном слое коры надпочечников может представлять собой отличный источник для образования органоидов.

G. Poli и соавт. смогли получить трехмерную органоидоподобную структуру из образцов надпочечников человеческого плода на разных сроках беременности. Диссоциированные клетки коры надпочечников и нейроэндокринные (хромаффинные) клетки спонтанно генерировали органоидоподобные образования, структурно и функционально схожие с надпочечниками плода как в кортикальной (экспрессия SF-1,StARи стероидогенных ферментов, способность секретировать кортизол), так и в нейроэндокринной (экспрессия хромогранина А, тирозингидроксилазы и нестин) частях [51]. В дальнейшем потребуются исследования для оценки долгосрочной жизнеспособности и функциональности этих фетальных (и постнатальных, когда таковые будут получены) органоидоподобных структур, особенно если они будут содержать популяцию стволовых клеток, способных восполнять функциональные клетки надпочечников.

## ЗАКЛЮЧЕНИЕ

И генная, и клеточная терапия обладают большим потенциалом, предлагая уникальные подходы для многих заболеваний.

Хотя подобные терапевтические достижения в сфере лечения патологии надпочечников разработаны меньше, чем в других областях эндокринологии, таких как лечение сахарного диабета, исследования последних двух десятилетий показывают, что существует высокий потенциал для лечения надпочечниковой недостаточности. К примеру, средняя продолжительность жизни пациентов с ВДКН 42 года, при этом наблюдаются инвалидизация и ограниченность социальной и трудовой активности. Описываемые терапевтические подходы предполагают восстановление эндогенной продукции гормонов, что может привести к излечению пациентов. Таким образом, в случае успеха эти методы могут радикально изменить стратегию лечения надпочечниковой недостаточности.

## ДОПОЛНИТЕЛЬНАЯ ИНФОРМАЦИЯ

Источники финансирования. Исследование выполнено с использованием средств государственного бюджета по госзаданию № 121030100031-0 от 02.03.2021.

Конфликт интересов. Авторы декларируют отсутствие явных и потенциальных конфликтов интересов, связанных с содержанием настоящей статьи.

Участие авторов. Глазова О.В. — по критерию 1 — основной исполнитель, создание концепции, подбор литературы; по критерию 2 — подготовка основного текста; Воронцова М.В. — по критерию 1 — научное руководство, по критерию 2 — внесение в рукопись важных правок; Шевкова Л.В. — по критерию 1 — вклад в дизайн текста, по критерию 2 — внесение в рукопись важных правок, оформление; Сакр Н. — по критерию 1 — вклад в дизайн текста, по критерию 2 — внесение в рукопись важных правок; Онянов Н.А. — по критерию 1 — вклад в концепцию текста, по критерию 2 — внесение в рукопись важных правок; Казиахмедова С.А. — по критерию 1 — вклад в концепцию текста, по критерию 2 — внесение в рукопись важных правок; Волчков П.Ю. — по критерию 1 — научное руководство, по критерию 2 — внесение в рукопись важных правок.

Все авторы одобрили финальную версию статьи перед публикацией, выразили согласие нести ответственность за все аспекты работы, подразумевающую надлежащее изучение и решение вопросов, связанных с точностью или добросовестностью любой части работы.

## References

[cit1] Yate R, Katugampola H, Cavlan D, et al. Adrenocortical Development, Maintenance, and Disease. Curr Top Dev Biol. 2013;106:239-312. doi: https://doi.org/10.1016/B978-0-12-416021-7.00007-9 24290352

[cit2] Steenblock Charlotte, Rubin de Celis Maria F., Delgadillo Silva Luis F., Pawolski Verena, Brennand Ana, Werdermann Martin, Berger Ilona, Santambrogio Alice, Peitzsch Mirko, Andoniadou Cynthia L., Schally Andrew V., Bornstein Stefan R. (2018). Isolation and characterization of adrenocortical progenitors involved in the adaptation to stress. Proceedings of the National Academy of Sciences.

[cit3] Nishimoto Koshiro, Harris Ruth B. S., Rainey William E., Seki Tsugio (2014). Sodium Deficiency Regulates Rat Adrenal Zona Glomerulosa Gene Expression. Endocrinology.

[cit4] Benc Damir, Icin Tijana, Pejakovic Sladjana, Bajkin Ivana, Prodanovic Jovana, Vukovic Bojan, Novakovic-Paro Jovanka, Tomic-Naglic Dragana, Zvezdin Biljana, Mitrovic Milena (2018). Glucocorticoid therapy and adrenal suppression. Medical review.

[cit5] Charmandari E, Nicolaides NC, Chrousos GP. Adrenal insufficiency. Lancet. 2014;383(9935):2152-2167. doi: https://doi.org/10.1016/S0140-6736(13)61684-0 24503135

[cit6] Kareva M A, Chugunov I S (2015). Federal clinical practice guidelines on the management of the patients presenting with congenital adrenal hyperplasia. Problems of Endocrinology.

[cit7] Buonocore Federica, Achermann John C. (2019). Primary adrenal insufficiency: New genetic causes and their long‐term consequences. Clinical Endocrinology.

[cit8] Turcu Adina F., Auchus Richard J. (2016). Novel treatment strategies in congenital adrenal hyperplasia. Current Opinion in Endocrinology, Diabetes & Obesity.

[cit9] Maharaj Avinaash, Maudhoo Ashwini, Chan Li F., Novoselova Tatiana, Prasad Rathi, Metherell Louise A., Guasti Leonardo (2019). Isolated glucocorticoid deficiency: Genetic causes and animal models. The Journal of Steroid Biochemistry and Molecular Biology.

[cit10] GOTOH HIDEO, SAGAI TOMOKO, HATA JUN-ICHI, SHIROISHI TOSHIHIKO, MORIWAKI KAZUO (2009). Steroid 21-Hydroxylase Deficiency in Mice*. Endocrinology.

[cit11] Riepe Felix G., Tatzel Stephan, Sippell Wolfgang G., Pleiss Jürgen, Krone Nils (2005). Congenital Adrenal Hyperplasia: The Molecular Basis of 21-Hydroxylase Deficiency in H-2aw18 Mice. Endocrinology.

[cit12] Hornstein S. R., Tajima T., Eisenhofer G., Haidan A., Aguilera G. (2018). Adrenomedullary function is severely impaired in 21‐hydroxylase‐deficient mice. The FASEB Journal.

[cit13] Tajima T, Okada T, Ma X-M, Ramsey W J, Bornstein S R, Aguilera G (2002). Restoration of adrenal steroidogenesis by adenovirus-mediated transfer of human cytochromeP450 21-hydroxylase into the adrenal gland of21-hydroxylase-deficient mice. Gene Therapy.

[cit14] Naiki Yasuhiro, Miyado Mami, Horikawa Reiko, Katsumata Noriyuki, Onodera Masafumi, Pang Songya, Ogata Tsutomu, Fukami Maki (2016). Extra-adrenal induction of Cyp21a1 ameliorates systemic steroid metabolism in a mouse model of congenital adrenal hyperplasia. Endocrine Journal.

[cit15] Perdomini M, Dos Santos C, Goumeaux C, Blouin V, Bougnères P (2017). An AAVrh10-CAG-CYP21-HA vector allows persistent correction of 21-hydroxylase deficiency in a Cyp21−/− mouse model. Gene Therapy.

[cit16] Markmann Sandra, De Bishnu P., Reid Jasmine, Jose Clarisse L., Rosenberg Jonathan B., Leopold Philip L., Kaminsky Stephen M., Sondhi Dolan, Pagovich Odelya, Crystal Ronald G. (2018). Biology of the Adrenal Gland Cortex Obviates Effective Use of Adeno-Associated Virus Vectors to Treat Hereditary Adrenal Disorders. Human Gene Therapy.

[cit17] Al-Dosari Mohammed S., Gao Xiang (2009). Nonviral Gene Delivery: Principle, Limitations, and Recent Progress. The AAPS Journal.

[cit18] Long Chengzu, Amoasii Leonela, Mireault Alex A., McAnally John R., Li Hui, Sanchez-Ortiz Efrain, Bhattacharyya Samadrita, Shelton John M., Bassel-Duby Rhonda, Olson Eric N. (2016). Postnatal genome editing partially restores dystrophin expression in a mouse model of muscular dystrophy. Science.

[cit19] Nelson Christopher E., Hakim Chady H., Ousterout David G., Thakore Pratiksha I., Moreb Eirik A., Rivera Ruth M. Castellanos, Madhavan Sarina, Pan Xiufang, Ran F. Ann, Yan Winston X., Asokan Aravind, Zhang Feng, Duan Dongsheng, Gersbach Charles A. (2016). In vivo genome editing improves muscle function in a mouse model of Duchenne muscular dystrophy. Science.

[cit20] Tabebordbar Mohammadsharif, Zhu Kexian, Cheng Jason K. W., Chew Wei Leong, Widrick Jeffrey J., Yan Winston X., Maesner Claire, Wu Elizabeth Y., Xiao Ru, Ran F. Ann, Cong Le, Zhang Feng, Vandenberghe Luk H., Church George M., Wagers Amy J. (2016). In vivo gene editing in dystrophic mouse muscle and muscle stem cells. Science.

[cit21] Yang Yang, Wang Lili, Bell Peter, McMenamin Deirdre, He Zhenning, White John, Yu Hongwei, Xu Chenyu, Morizono Hiroki, Musunuru Kiran, Batshaw Mark L, Wilson James M (2016). A dual AAV system enables the Cas9-mediated correction of a metabolic liver disease in newborn mice. Nature Biotechnology.

[cit22] Thomas Clare E., Ehrhardt Anja, Kay Mark A. (2003). Progress and problems with the use of viral vectors for gene therapy. Nature Reviews Genetics.

[cit23] Chen Shuai, Du Kechen, Zou Chunlin (2020). Current progress in stem cell therapy for type 1 diabetes mellitus. Stem Cell Research & Therapy.

[cit24] Thomas Michael, Northrup S. Robert, Hornsby Peter J. (2004). Adrenocortical tissue formed by transplantation of normal clones of bovine adrenocortical cells in scid mice replaces the essential functions of the animals' adrenal glands. Nature Medicine.

[cit25] Thomas Michael, Hornsby Peter J (2002). Transplantation of primary bovine adrenocortical cells into scid mice. Molecular and Cellular Endocrinology.

[cit26] Thomas Michael, Wang Xiangdong, Hornsby Peter J. (2004). Human adrenocortical cell xenotransplantation: Model of cotransplantation of human adrenocortical cells and 3T3 cells in scid mice to form vascularized functional tissue and prevent adrenal insufficiency. Xenotransplantation.

[cit27] Popnikolov Nikolay K., Hornsby Peter J. (2017). Subcutaneous Transplantation of Bovine and Human Adrenocortical Cells in Collagen Gel in scid Mice. Cell Transplantation.

[cit28] Dunn James C.Y., Chu Yinting, Lam Mandy M., Wu Benjamin M., Atkinson James B., McCabe Edward R. (2004). Adrenal cortical cell transplantation. Journal of Pediatric Surgery.

[cit29] Zupekan Tatiana, Dunn James C.Y. (2011). Adrenocortical cell transplantation reverses a murine model of adrenal failure. Journal of Pediatric Surgery.

[cit30] Allen Robert A., Seltz Lara M., Jiang Hongbin, Kasick Rena T., Sellaro Tiffany L., Badylak Stephen F., Ogilvie Jennifer B. (2010). Adrenal Extracellular Matrix Scaffolds Support Adrenocortical Cell Proliferation and FunctionIn Vitro. Tissue Engineering Part A.

[cit31] Balyura Mariya, Gelfgat Evgeny, Ehrhart-Bornstein Monika, Ludwig Barbara, Gendler Zohar, Barkai Uriel, Zimerman Baruch, Rotem Avi, Block Norman L., Schally Andrew V., Bornstein Stefan R. (2015). Transplantation of bovine adrenocortical cells encapsulated in alginate. Proceedings of the National Academy of Sciences.

[cit32] Teebken Omke Enno, Scheumann Georg Friedrich Wilhelm (2004). DIFFERENTIATED CORTICOSTEROID PRODUCTION AND REGENERATION AFTER SELECTIVE TRANSPLANTATION OF CULTURED AND NONCULTURED ADRENOCORTICAL CELLS IN THE ADRENALECTOMIZED RAT1. Transplantation.

[cit33] Mariniello K, Ruiz-Babot G, McGaugh EC, et al. Stem Cells, Self-Renewal, and Lineage Commitment in the Endocrine System. Front Endocrinol (Lausanne). 2019;10:772. doi: https://doi.org/10.3389/fendo.2019.00772 PMC6856655 31781041

[cit34] Freedman Bethany D., Kempna Petra Bukovac, Carlone Diana L., Shah Manasvi S., Guagliardo Nick A., Barrett Paula Q., Gomez-Sanchez Celso E., Majzoub Joseph A., Breault David T. (2013). Adrenocortical Zonation Results from Lineage Conversion of Differentiated Zona Glomerulosa Cells. Developmental Cell.

[cit35] Dunn James C.Y., Chu Yinting, Qin Harry H., Zupekan Tatiana (2009). Transplantation of Adrenal Cortical Progenitor Cells Enriched by Nile Red. Journal of Surgical Research.

[cit36] Thomas Michael, Suwa Tetsuya, Yang Lianqing, Zhao Lifang, Hawks Christina L., Hornsby Peter J. (2002). Cooperation of hTERT, SV40 T Antigen and Oncogenic Ras in Tumorigenesis: A Cell Transplantation Model Using Bovine Adrenocortical Cells. Neoplasia.

[cit37] Dimitrioglou Nikos, Kanelli Maria, Papageorgiou Efstathia, Karatzas Theodore, Hatziavramidis Dimitris (2019). Paving the way for successful islet encapsulation. Drug Discovery Today.

[cit38] Takahashi Kazutoshi, Yamanaka Shinya (2006). Induction of Pluripotent Stem Cells from Mouse Embryonic and Adult Fibroblast Cultures by Defined Factors. Cell.

[cit39] Crawford P A, Sadovsky Y, Milbrandt J (2015). Nuclear receptor steroidogenic factor 1 directs embryonic stem cells toward the steroidogenic lineage. Molecular and Cellular Biology.

[cit40] Yazawa Takashi, Kawabe Shinya, Inaoka Yoshihiko, Okada Reiko, Mizutani Tetsuya, Imamichi Yoshitaka, Ju Yunfeng, Yamazaki Yukiko, Usami Yoko, Kuribayashi Mayu, Umezawa Akihiro, Miyamoto Kaoru (2010). Differentiation of mesenchymal stem cells and embryonic stem cells into steroidogenic cells using steroidogenic factor-1 and liver receptor homolog-1. Molecular and Cellular Endocrinology.

[cit41] Sonoyama Takuhiro, Sone Masakatsu, Honda Kyoko, Taura Daisuke, Kojima Katsutoshi, Inuzuka Megumi, Kanamoto Naotetsu, Tamura Naohisa, Nakao Kazuwa (2012). Differentiation of Human Embryonic Stem Cells and Human Induced Pluripotent Stem Cells into Steroid-Producing Cells. Endocrinology.

[cit42] Gondo Shigeki, Yanase Toshihiko, Okabe Taijiro, Tanaka Tomoko, Morinaga Hidetaka, Nomura Masatoshi, Goto Kiminobu, Nawata Hajime (2004). SF-1/Ad4BP transforms primary long-term cultured bone marrow cells into ACTH-responsive steroidogenic cells. Genes to Cells.

[cit43] Tanaka Tomoko, Aoyagi Chikao, Mukai Kuniaki, Nishimoto Koshiro, Kodama Shohta, Yanase Toshihiko (2020). Extension of Survival in Bilaterally Adrenalectomized Mice by Implantation of SF-1/Ad4BP-Induced Steroidogenic Cells. Endocrinology.

[cit44] Gondo Shigeki, Okabe Taijiro, Tanaka Tomoko, Morinaga Hidetaka, Nomura Masatoshi, Takayanagi Ryoichi, Nawata Hajime, Yanase Toshihiko (2008). Adipose Tissue-Derived and Bone Marrow-Derived Mesenchymal Cells Develop into Different Lineage of Steroidogenic Cells by Forced Expression of Steroidogenic Factor 1. Endocrinology.

[cit45] Yazawa Takashi, Mizutani Tetsuya, Yamada Kazuya, Kawata Hiroko, Sekiguchi Toshio, Yoshino Miki, Kajitani Takashi, Shou Zhangfei, Umezawa Akihiro, Miyamoto Kaoru (2006). Differentiation of Adult Stem Cells Derived from Bone Marrow Stroma into Leydig or Adrenocortical Cells. Endocrinology.

[cit46] Yazawa Takashi, Inanoka Yoshihiko, Mizutani Tetsuya, Kuribayashi Mayu, Umezawa Akihiro, Miyamoto Kaoru (2009). Liver Receptor Homolog-1 Regulates the Transcription of Steroidogenic Enzymes and Induces the Differentiation of Mesenchymal Stem Cells into Steroidogenic Cells. Endocrinology.

[cit47] Yazawa Takashi, Inaoka Yoshihiko, Okada Reiko, Mizutani Tetsuya, Yamazaki Yukiko, Usami Yoko, Kuribayashi Mayu, Orisaka Makoto, Umezawa Akihiro, Miyamoto Kaoru (2010). PPAR-γ Coactivator-1α Regulates Progesterone Production in Ovarian Granulosa Cells with SF-1 and LRH-1. Molecular Endocrinology.

[cit48] Wei X., Peng G., Zheng S., Wu X. (2012). Differentiation of umbilical cord mesenchymal stem cells into steroidogenic cells in comparison to bone marrow mesenchymal stem cells. Cell Proliferation.

[cit49] Ruiz-Babot Gerard, Balyura Mariya, Hadjidemetriou Irene, Ajodha Sharon J., Taylor David R., Ghataore Lea, Taylor Norman F., Schubert Undine, Ziegler Christian G., Storr Helen L., Druce Maralyn R., Gevers Evelien F., Drake William M., Srirangalingam Umasuthan, Conway Gerard S., King Peter J., Metherell Louise A., Bornstein Stefan R., Guasti Leonardo (2018). Modeling Congenital Adrenal Hyperplasia and Testing Interventions for Adrenal Insufficiency Using Donor-Specific Reprogrammed Cells. Cell Reports.

[cit50] Lancaster Madeline A., Huch Meritxell (2019). Disease modelling in human organoids. Disease Models & Mechanisms.

[cit51] Poli Giada, Sarchielli Erica, Guasti Daniele, Benvenuti Susanna, Ballerini Lara, Mazzanti Benedetta, Armignacco Roberta, Cantini Giulia, Lulli Matteo, Chortis Vasileios, Arlt Wiebke, Romagnoli Paolo, Vannelli Gabriella Barbara, Mannelli Massimo, Luconi Michaela (2018). Human fetal adrenal cells retain age‐related stem‐ and endocrine‐differentiation potential in culture. The FASEB Journal.

